# Diffusion Tensor Tractography Studies for Causes of Dysphagia After Stroke: A Systematic Review

**DOI:** 10.3390/brainsci15090925

**Published:** 2025-08-27

**Authors:** Woo-Hyuk Jang, Seon-Hee Lee, Sang-Hyeok Lee

**Affiliations:** 1 Department of Occupational Therapy, College of Health Science, Kangwon National University, Samcheok-si 25949, Republic of Korea; wlqtksek@hanmail.net; 2 Department of Occupational Therapy, Graduate School, Kangwon National University, Samcheok-si 25949, Republic of Korea; lamb0317@naver.com

**Keywords:** stroke, diffusion tensor tractography, swallowing, dysphagia, systematic review

## Abstract

**Background/Objectives**: This systematic review aimed to investigate the causes of dysphagia after stroke through diffusion tensor tractography (DTT) studies. **Methods**: This review used databases such as Google Scholar, PubMed, and ScienceDirect. Keywords related to stroke, dysphagia, and diffusion tensor tractography were utilized. Seven studies were selected and analyzed. **Results**: The analysis identified that damage to the corticobulbar tract (CBT) was the most frequently reported cause of dysphagia. Additionally, some studies suggested that damage to the vestibulospinal tract (VST) and the core vestibular pathway (CVP) contributed to dysphagia. Moreover, a significant negative correlation was found between dysphagia severity and key DTT-derived metrics, such as lower fractional anisotropy (FA) and tract volume (TV), indicating that reduced FA and TV values are associated with more severe dysphagia symptoms. **Conclusions**: DTT provides valuable insights into the neural mechanisms underlying dysphagia after stroke. Identifying the affected tracts can help diagnose dysphagia more accurately and develop targeted rehabilitation strategies.

## 1. Introduction

Stroke is a neurological deficit caused by damage to the brain and the central nervous system, and it can be broadly categorized into two types: infarction and hemorrhage [1,2]. Infarction is the damage to brain neural circuits caused by the closure of blood vessels, while hemorrhage is the damage to nerve cells caused by the rupture of blood vessels [3]. As a result, stroke can cause various problems, including plegia, language, cognition, and perception [4]. In addition to these complications, dysphagia is a common complication [5]. Dysphagia refers to difficulty swallowing and, on average, occurs in about 42–67% of stroke patients [6], with about half of all patients developing it after an acute stroke [7]. Stroke causes various symptoms depending on the type and area of the damaged blood vessel, and the middle cerebral artery (MCA), which is the most frequently damaged blood vessel, supplies blood to various areas of the brain, causing functional problems related to the frontal, temporal, and parietal lobes [5]. Therefore, in addition to dysphagia, different symptoms, such as plegia and neglect, may occur [5]. Particularly, if an infarction occurs in the lateral medulla, it is called ‘lateral medullary syndrome’ or ‘Wallenberg’s syndrome’ [8]. The lateral medulla contains the main nuclei of swallowing that trigger and control the swallowing reflex, so when it is damaged, dysphagia occurs at a higher rate than in other areas [9]. Dysphagia, which causes difficulty swallowing, can lead to aspiration of food or saliva, which can increase the risk of complications such as pneumonia [7]. In addition, half of people with dysphagia rely on a nasogastric tube (NGT) as an alternative to oral intake due to the persistence of symptoms [10]. Dysphagia adversely affects stroke recovery by causing problems with activities of daily living (ADL), such as malnutrition and increased length of hospitalization [7,10], as well as decreased quality of life and depression arising from this dependency and fear of a poor prognosis [11,12,13]. Due to these various causes and symptoms, dysphagia is a crucial factor to consider in stroke patients [14]. Therefore, evaluating for the presence of dysphagia is essential, and tests such as videofluoroscopic swallowing study (VFSS), fiberoptic endoscopic evaluation of swallowing (FEES), and the 3-ounce water-swallow test are performed to confirm it [7,14].

Various studies have been conducted using diffusion-weighted images (DWI) to investigate the causes of dysphagia [15,16,17,18,19]. DWI was developed in the 1980s and is a magnetic resonance imaging (MRI) technique that uses the diffusive motion of water molecules to image fine structures in the brain [20]. First, Lee et al. (2020) reported that bilateral lesions of the basal ganglia, corona radiata, and internal capsule were critical factors in the cause and severity of early dysphagia in 137 patients with acute ischemic stroke [15]. Fandler et al. (2017) reported in a study of 332 patients with cerebellar infarction that dysphagia can occur in significant numbers in patients with cerebellar infarction and that additional damage to the pons or severe damage to white matter may increase the risk of dysphagia [16]. Flowers et al. (2017), on the other hand, reported that the main predictors of dysphagia were medullary, insular, and pontine lesions in 160 patients with acute ischemic stroke [17]. Wilmskoetter et al. (2019) found that in 68 patients with middle cerebral artery stroke, dysphagia symptoms were mainly seen in cortical regions, subcortical regions, and white matter tracts damage in the right hemisphere [18]. Fandler et al. (2018) reported in 243 patients with small subcortical infarct (RSSI) that moderate-to-severe dysphagia in RSSI patients was primarily due to bilateral pyramidal tract damage or a combination of bilateral RSSI damage and contralateral vascular lesions [19]. This ability of DWI to image different tissues in the brain has led to several reports on the location of dysphagia [19].

**Figure 1:**
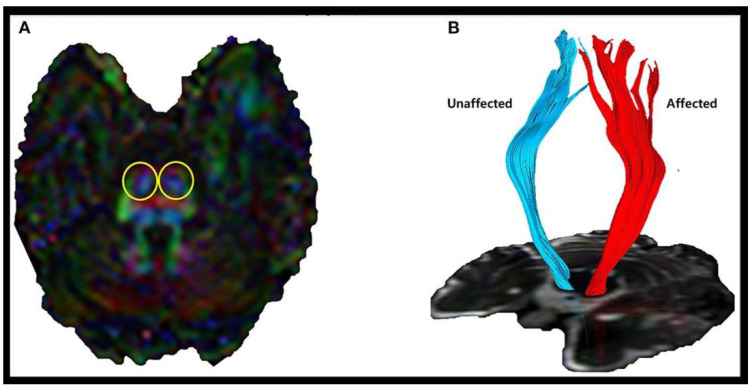
Example of diffusion tensor tractography (DTT). (**A**) Yellow circle shows regions of interest (lower anterior pons). (**B**) Corticospinal tract (CST) visualized by DTT [22]. Copyright by Kim et al. (2022) [22].

Diffusion tensor tractography is a technique that allows visualization of damaged brain neural tract connections and can measure various metrics such as fractional anisotropy (FA), tract volume (TV), apparent diffusion coefficient (ADC), and mean diffusivity (MD) of neural tracts [21,22] (Figure 1). FA is an indicator of the directionality of neural tracts and the alignment of their organizational structure [23]. Next, TV is a measure of the total volume of neural fiber bundles and is used to assess the overall size of a particular neural tract [24]. ADC is a diffusion metric that shows how diffuse a neural tract is, and MD is the mean diffusivity [25]. When a neural tract is damaged, FA and TV values decrease while ADC and MD values increase [24,25]. Therefore, DTT has the main advantage of visualizing, quantifying, and evaluating the structural integrity, connectivity, and spatial organization of an entire neural tract using the above metrics [19]. Furthermore, in viewing neural tract connections using DTT equipment, regions of interest (ROI) are established, representing specific areas of interest [26]. Specifically, they can be divided into seed ROI and target ROI, where the seed ROI is the starting point of the tract analysis process, and the target ROI is the final destination of the connections in the tract that started in the seed ROI [27]. Due to the usefulness of DTT, its application is expanding beyond stroke and brain damage to include dementia and Parkinson’s disease [28]. In addition, recent research has focused on the spinal cord [29].

The importance of identifying the etiology of dysphagia symptoms and the effectiveness of interventions urged a review study to identify the etiology of dysphagia using various instruments [30]. However, the review only focused on DWI and did not identify constructed connections and detailed indices, such as FA, TV, and MD [30]. Hence, it is necessary to use DTT, which can visualize neural connections and quantify multiple visual images of neural connections as indices, to identify in detail the neural tracts that cause dysphagia.

Accordingly, this study aimed to systematically review the literature utilizing DTT to identify the specific neural tracts implicated in after-stroke dysphagia by examining the findings on their structural changes, as measured by metrics such as FA, TV, and MD.

## 2. Methods

This systematic review was conducted in accordance with the guidelines outlined in the Cochrane Handbook of Systematic Reviews of Interventions [31] and adhered to the Preferred Reporting Items for Systematic Reviews and Meta-Analyses (PRISMA 2020) guidelines [32].

The literature search was conducted from December 2023 to March 2025. The databases used in this study were Google Scholar, PubMed, and ScienceDirect, and the following keywords were used in the search: ‘human’ AND ‘stroke’, ‘Diffusion Tensor Imaging’ OR ‘Diffusion Tensor Tractography’, ‘swallowing disorder’ OR ‘lateral medullary syndrome’, and ‘dysphagia’. Twenty-six articles were retrieved as a result of the literature search.

**Figure 2:**
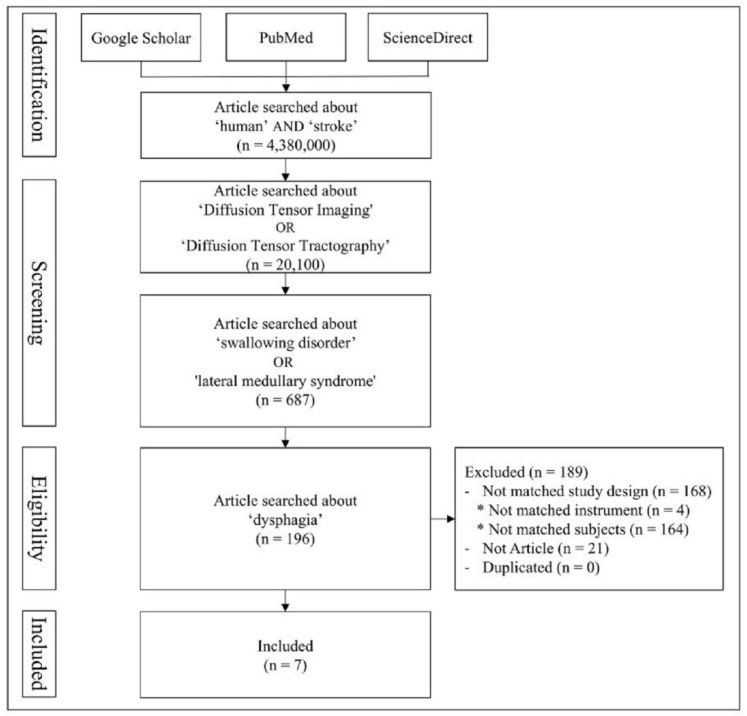
Flow diagram of this study.

Articles were eligible if they (a) were written in English, (b) were a full-text paper, (c) involved humans, (d) were original research published in scientific journals, (e) involved patients with a diagnosis of dysphagia because of stroke or lateral medullary syndrome, and (f) used DTT. Therefore, we excluded (a) non-English-written papers, (b) articles on non-full text papers, (c) systematic and narrative reviews and meta-analyses, (d) those without dysphagia the objective of stroke or lateral medullary syndrome and dysphagia, and (e) those with non-use of DTT. After excluding studies that met the exclusion criteria and duplicate searches, seven articles were selected (Figure 2).

## 3. Results

### 3.1. Four Corticobulbar Tract Studies on Patients of Dysphagia

**Figure 3:**
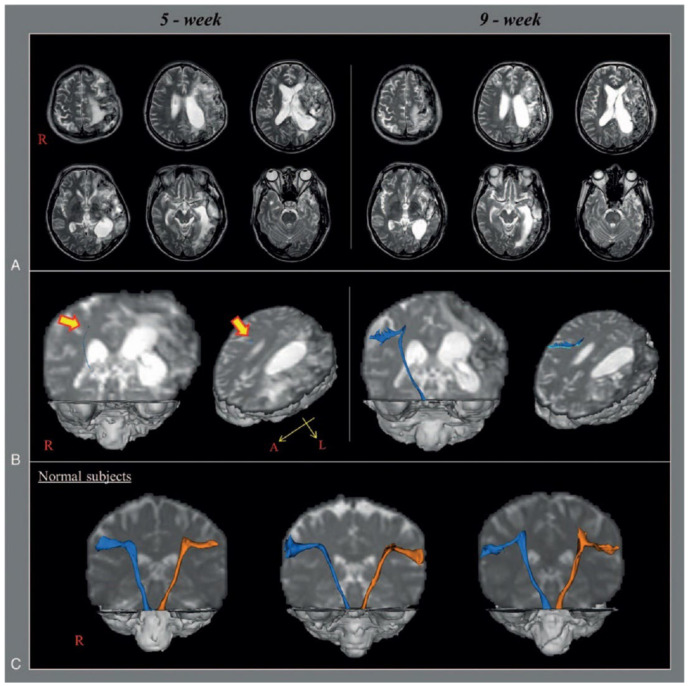
Anatomical structure of CBT captured using DTT [33]. (**A**) T2-weighted brain magnetic resonance (MR) images at 5 and 9 weeks after onset show leukomalactic lesions in left middle cerebral artery territory. (**B**) Diffusion tensor tractography (DTT) for the corticobulbar tract (CBT). On 5-week DTT, the right CBT is discontinued at the subcortical white matter and shows severe narrowing compared with those of normal subjects, whereas the left CBT is not reconstructed. By contrast, on 9-week DTT, the right CBT is extended to the cerebral cortex with thickening, whereas the left CBT is still not reconstructed. (**C**) Reconstructed CBT of normal subjects (55-year-old man, 57-year-old man, and 60-year-old man), blue arrow: right CBT, orange arrow: left CBT. Copyright by Jang et al. (2017) [33].

Jang et al. (2017) studied the neural tract causing dysphagia in a stroke patient (*n* = 1, 59 years old, male, left middle cerebral artery infarction) using diffusion tensor tractography [33]. The study subject was wearing a Levin tube due to severe dysphagia after a stroke [33] (Figure 3). DTT was performed at 5 and 9 weeks post stroke, and the results were compared to those of normal individuals of the same gender and age (control group, *n* = 3). The DTT results at week 5 revealed that the left CBT was not analyzed due to severe damage, and the right CBT was only marginally analyzed due to severe narrowing at the subcortical white matter level. However, the patient showed improvement in dysphagia at week 9, so the Levin tube was removed, and the patient started to eat normally. In addition, the DTT at week 9 showed that the left CBT had not been reconstructed in the same way as it was at week 5, but the right CBT was smoothly reconstructed, extending into the cerebral cortex. Thus, CBT is significantly involved in the pathogenesis of dysphagia; particularly, the recovery of the right CBT is associated with the recovery of dysphagia symptoms, even though the left CBT was not reconstructed. Limitations of this study include the potential for false-negative results due to crossing fibers and partial volume effects imaged using DTT. Second, although the reconstruction of CBT was identified, the parameters of DTT, such as fractional anisotropy, tract volume, and mean diffusivity, were not specified. Finally, the mechanism by which dysphagia improved after restoration of unilateral CBT was not explained.

Jang et al. (2020) examined the prognostic value of CBT parameters (fractional anisotropy and tract volume) and its prediction of dysphagia using DTT in stroke patients (experimental group, *n* = 42, intracerebral hemorrhage) and normal individuals (control group, *n* = 22) [34]. The experimental group was divided into A group (*n* = 10): removal of nasogastric tube in the acute stage (within 2 days after onset); B group (*n* = 27): NGT removal within 6 months after onset; and C group (*n* = 5): NGT removal more than 6 months after onset. DTT was taken within 6 weeks of onset and compared to the DTT results of the control group. Compared to the control group, group A had a significant decrease in FA values in the affected side (i.e., CBT only), and group B showed a significant decrease in both FA and TV values in the affected side. However, the C group showed a significant decrease in both FA and TV values of both sides of CBT. A moderate negative correlation (r = 0.430; *p* < 0.05) was also found, illustrating that the higher the TV value of the affected side CBT in the B group, the shorter the duration of NGT wearing. Another finding presented that damage to the CBT of both sides made it challenging to remove the NGT within 6 months and that recovery was possible within 6 months if only the CBT of the affected side was damaged. Consequently, the current study verified that the identification of CBT parameters (FA and TV) can help recognize the tract responsible for dysphagia in stroke patients and prognostic prediction for recovery. Some limitations of Jang et al.’s (2020) study are as follows: (1) the small number of study subjects, affecting the generalizability of the results; (2) the presence of subjects with IVH in the experimental group, resulting in an imbalance in the homogeneity of the subjects in the experimental group; (3) the difference in the number of subjects in the two groups, leading to an imbalance in the number of subjects in each subgroup of the experimental group; (4) the mean diffusivity and apparent diffusion coefficient metrics of the neural tracts were not mentioned; and (5) there was no explanation of the mechanism by which dysphagia improved with unilateral CBT [34].

Jang et al. (2020) studied the prognostic value of CBT parameters and its prediction of dysphagia symptoms using DTT in stroke patients (experimental group, *n* = 20, lateral medullary infarction (LMI)) and normal individuals (control group, *n* = 20) [35]. The experimental group comprised patients who had experienced or were currently wearing an NGT due to dysphagia post infarction. The experimental group was divided into two subgroups: A group (*n* = 16): NGT removal within 6 months of onset; and B group (*n* = 4): NGT removal more than 6 months after onset. DTT of both groups was taken within 6 weeks of onset and compared with the results of the control group. The results revealed that the FA values of CBT in the affected hemisphere were significantly lower in group B. Moreover, the TV values of CBT in both hemispheres were significantly reduced in both groups compared to the control group, although there was no significant difference between groups A and B. Furthermore, the damage was severe enough that the CBT on the affected side was not reconstructed in three out of four subjects in group B. This suggests that LMI-induced dysfunction in the affected hemisphere may be related to the lack of reconstruction. This study demonstrated that the extent and recovery of LMI-induced dysphagia are associated with CBT damage and confirmed that DTT can be used for the prognostic prediction of dysphagia from the early stages of damage. Limitations of this study are as follows: (1) the small number of subjects in this study, which makes it challenging to generalize the findings; (2) the imbalance in the number of subjects in each subgroup of the experimental group; (3) the analysis of the underlying fiber architecture, which may not be complete in regions of fiber complexity and crossing; and (4) the MD and ADC values of the neural tracts were not mentioned.

Wang et al. (2023) investigated the treatment effect of post-stroke dysphagia with repetitive transcranial magnetic stimulation (rTMS) using DTT in stroke patients (*n* = 61) [36]. The FA of CBT of 61 subjects was measured using DTT [36]. In addition, 31 subjects with high CBT integrity were divided into three groups and given the following rTMS treatments: 5 Hz rTMS (*n* = 11) or 1 Hz rTMS (*n* = 10) and a control group (Sham rTMS (*n* = 10). Then, 30 participants with low CBT integrity were equally divided into three groups: 5 Hz rTMS (*n* = 10) or 1 Hz rTMS (*n* = 10) and control (Sham rTMS, *n* = 10). For rTMS, the higher the number of Hz, the stronger the stimulation; for Sham rTMS, no current was delivered, but the same frequency, stimulation duration, and sound environment as 5 Hz was used. The videofluoroscopic swallowing study (VFSS), the standardized swallowing assessment (SSA), penetration aspiration scale (PAS), and dysphagia outcome severity scale (DOSS) were used to assess dysphagia. The results indicated that the high CBT integrity group showed a significant recovery of dysphagia symptoms at 5 Hz and 1 Hz compared to Sham, without significant difference between 5 Hz and 1 Hz. However, the low-CBT-integrity group showed recovery of dysphagia symptoms only at 5 Hz. Additionally, significant treatment effects on SSA, PAS, and DOSS were found in both high- and low-CBT-integrity groups, implying that high-frequency rTMS intervention is a more effective intervention for the recovery of dysphagia symptoms due to severe CBT impairment. The paper’s limitations include the lack of long-term follow-up of dysphagia symptoms; the small number of studied subjects; the lack of explicit focus on addressing the association between CBT and dysphagia; and the use of only FA measures of CBT, excluding MD and TV.

### 3.2. Two Vestibulospinal Tract Studies on Patients of Dysphagia

Jang et al. (2020) studied the association between the vestibulospinal tract and central vestibular disorder symptoms causing dysphagia using DTT in a stroke patient (*n* = 1, 56 years old, male, right lateral medullary syndrome due to right posterior inferior cerebellar artery infarction) [37]. DTT was taken on average at 2 weeks post onset and compared to the results of six normal individuals of similar age. The results showed no damage to the bilateral medial vestibulospinal tract (VST) and left lateral VST of the experimental group and no significant differences in FA or MD from the control group. However, the right lateral VST of the experimental group was not reconstructed after damage to the extent that the FA and MD metrics was difficult to measure. Central vestibular disorder symptoms persisted at the 6-week post-injury assessment. This study demonstrated that central vestibular disorder symptoms in patients with lateral medullary syndrome due to PICA infarction resulted from lateral VST damage. The study’s limitations, which affected the generalizability of the results, are as follows: the case report study covered narratives specific to the study subjects; only a small number of subjects were involved in the study; the small size of the vestibular nuclei made it impossible to precisely position the ROI; the fiber tracts could be underestimated; dysphagia, one of the core symptoms of central vestibular disorder, was not discussed in detail; and the TV and ADC values of the nerve tracts were not specified.

**Figure 4:**
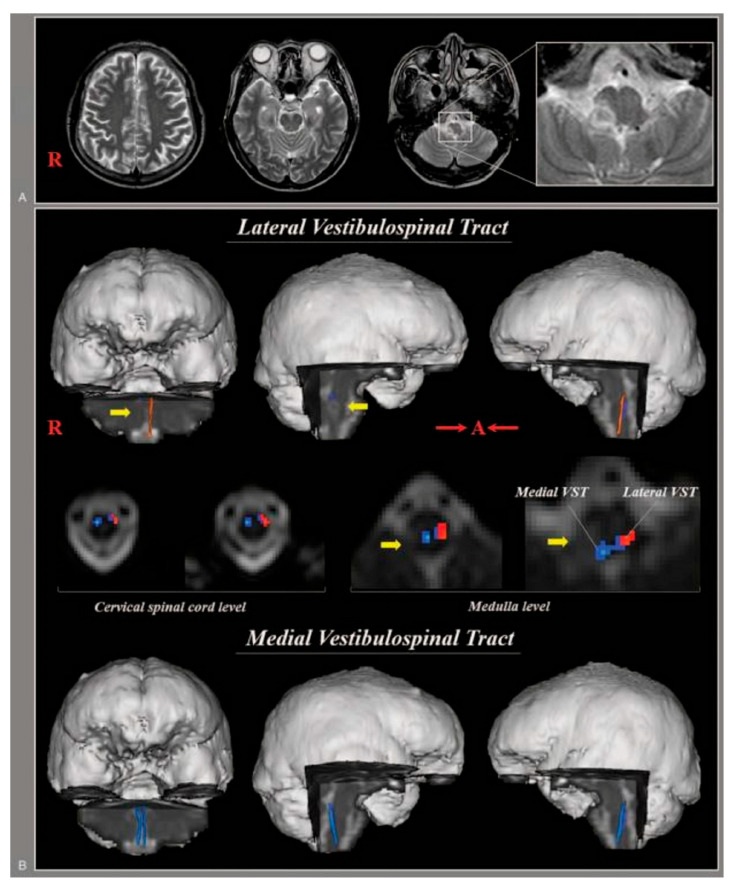
Anatomical structure of VST captured using DTT [38]. (**A**) Brain MR images at 2 weeks after onset show an infarction in the right dorsolateral medulla. (**B**) Results of diffusion tensor tractography (DTT). DTTs for both the medial vestibulospinal tract (VST) and the left lateral VST originate from the pontine vestibular nuclei and terminate at upper cervical cord. By contrast, DTT for the right lateral VST is not reconstructed between the lateral vestibular nuclei in pons and the upper cervical cord. Copyright by Jang et al. (2020) [38].

Jang et al. (2020) studied stroke patients (experimental group, *n* = 7, lateral medullary syndrome due to dorsolateral medullary infarction) and normal individuals (control group, *n* = 10) using DTT to compare parameters according to medial and lateral vestibulospinal tract damage and to determine the association of central vestibular disorder with dysphagia [38] (Figure 4). The experimental group presented with typical central vestibular disorders (dysphagia: *n* = 6, vertigo: *n* = 6, ataxia: *n* = 4, dysarthria: *n* = 3). DTT was performed at an average of 2 weeks after onset, and FA, MD, and TV of the corticospinal tracts (CST) were measured together for comparative analysis of medial and lateral VST and motor function and compared with DTT results of 10 control subjects. The results showed that in the CST and medial VST, the FA, MD, and TV in both sides were not significantly different from the control group, but in the lateral VST, FA values in the non-affected side were significantly reduced compared to the control group, and both FA and TV values in the affected side were significantly reduced compared to the control group. These findings suggest that metric analysis of lateral VST using DTT can help assess central vestibular sign symptoms such as dysphagia in patients with lateral medullary syndrome and in planning future interventions. Some limitations of this study are as follows: the small number of subjects in the study; the small size of the vestibular nuclei, which prevented the researchers from precisely positioning the ROI; the lack of data on long-term follow-up; the researchers may have underestimated or overestimated the fiber tracts of the VST; and the researchers could not reconstruct the full length of the lateral VST.

### 3.3. One Core Vestibular Pathway Studies on Patients of Dysphagia

**Figure 5:**
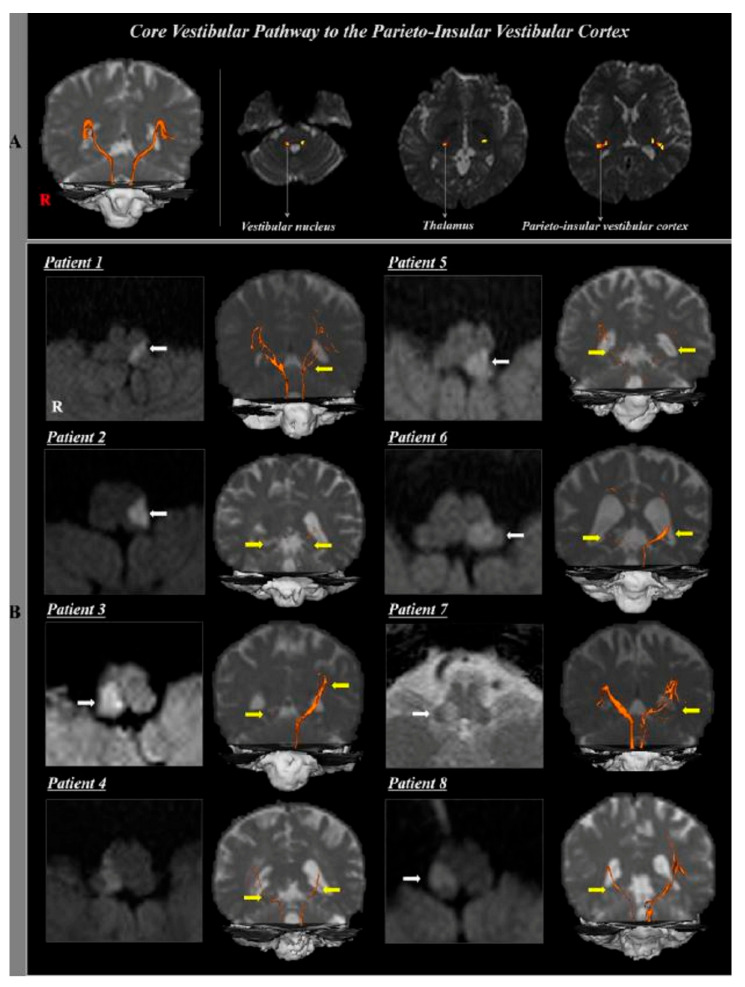
Anatomical structure of CVP captured using DTT [39]. (**A**) Core vestibular pathway to the PIVC in normal control subject (53-year-old male) (**B**) Computed tomography (CT) and diffusion tensor tractography (DTT) of the core vestibular pathway to the PIVC in eight patients with dorsolateral medullary infarct (white arrow).; bilateral or unilateral core vestibular pathway to the PIVC were injured due to dorsolateral medullary infarct (yellow arrow). Copyright by Yeo et al. (2018) [39].

Yeo et al. (2018) used DTT in stroke patients (experimental group, *n* = 8, lateral medullary syndrome) and healthy individuals (control group, *n* = 10) to study the association between damage to the core vestibular pathway (connecting the parieto-insular vestibular cortex (PIVC) and vestibular nuclei) and symptoms of central vestibular disorder [39] (Figure 5). The experimental group demonstrated symptoms of central vestibular disorder (vertigo [*n* = 7], dysphagia [*n* = 6], ataxia [*n* = 5], dysarthria [*n* = 3]). The DTT results for the core vestibular pathway were compared between the two groups. The results presented no significant difference between the two groups in FA and MD of the core vestibular pathway, but both the affected side and non-affected side of the experimental group significantly decreased in tract volume compared to the control group. The results confirm that damage to the core vestibular pathway is highly associated with dysphagia. Some limitations of the research include (1) the small number of subjects, making it difficult to generalize the results; (2) the tract volume of the fiber tract of the core vestibular pathway, which may have been underestimated or overestimated; (3) the contralateral vestibular pathway on the non-affected side that could not be reconstructed; (4) the vestibular nuclei area for seed regions of interest, which was too small to define accurately; and (5) the lack of explanation of the degree of reconstruction of the left and right tracts and the difference between moderate and sub-severe symptoms of central vestibular disorder.

## 4. Discussion

This study systematic reviewed seven articles, focusing on the type of tracts that cause dysphagia in stroke patients using diffusion tensor tractography (Table 1).

First, regarding the tracts involved in causing dysphagia, the corticobulbar tract (CBT) was found to be the most common among the four studies. Specifically, in the case of CBT, cases of dysphagia symptoms improved with the recovery of the right CBT alone, even when the left CBT was not recovered [33,34]. This outcome can be explained by the ‘bilateral cortical representation’ of Broadbent’s law from previous studies [40]. This finding is consistent with previous research suggesting that muscles involved in survival, such as breathing and swallowing, are controlled by the bilateral cerebral cortex so that damage to one side of the tract does not directly result in complete loss of function [40,41].

Another tract implicated in the development of dysphagia was the vestibulospinal tract, reported in two studies [37,38]. On the other hand, one study considered the core vestibular pathway as a factor [39]. Injury to the VST has been associated with multiple symptoms of central vestibular disorders (e.g., vertigo, ataxia, and aphasia), particularly showing a strong correlation with dysphagia in patients with lateral medullary syndrome [37,38,39]. Similarly, damage to the CVP was also frequently associated with central vestibular disorders, including dysphagia [39].

Specifically, in the reviewed DTT parameters, fractional anisotropy appeared as the most commonly reported [34,35,36,37,38,39], followed by tract volume [34,35,38] and mean diffusivity [37,38,39]. Furthermore, one study demonstrated the neural tract injury by comparing images directly with those of healthy controls, without employing specific parameters [33]. Additionally, correlations between changes in these parameters and dysphagia severity were identified, with FA and TV values of the CBT and VST exhibiting a negative correlation, indicating that lower FA and TV values reflected greater tract damage. Moreover, certain studies went beyond identifying tracts responsible for dysphagia and evaluated prognostic predictions of dysphagia recovery, such as prolonged use of nasogastric tube correlated with the severity of neural tract injury [34,35].

Next, in terms of the study subjects, three studies included patients with lateral medullary syndrome due to stroke [37,38,39], two studies examined patients with infarction [33,35], one study investigated patients with cerebral hemorrhage [34], and one study involved stroke patients without differentiating infarction from hemorrhage or patients with complex brain injury. DTT imaging was typically performed within an average of 14 days after stroke onset, with all studies conducting initial imaging no later than six weeks post onset.

Regarding the study designs, cohort studies were the most frequent, as reported in four papers [34,35,38,39], followed by two case studies [33,37], and one pilot study [36]. Identified limitations across these studies included incomplete reporting of certain measurable parameters through DTT [33,35,36,37], a small sample size that limited generalizability [35,36,37], potential false-negative results in regions where neural fibers cross intricately due to technical limitations of DTT equipment [35,36,37], absence of long-term follow-up assessments [36,37], and difficulties in accurately defining regions of interest due to the small size of certain neural nuclei [37]. Additionally, this systematic review has several limitations. First, the types of stroke and lesion locations varied across the studies, highlighting the need to consider the specific impact of the injury site on the results. Second, differences existed in the defined regions of interest (ROI) and the timing of scans among the studies. Finally, with three different study designs identified across the seven articles, we suggest that future research should apply DTT to dysphagia patients using more varied study designs and including a wider range of stroke types.

Nevertheless, this review identified major neural tracts responsible for dysphagia using DTT. Furthermore, it emphasized the potential to analyze the correlation between various parameters derived from DTT and the severity of dysphagia as well as to predict recovery outcomes. Through this paper, was confirmed that DTT can play a crucial role not only in identifying the neural pathways responsible for swallowing difficulties after stroke but also in quantitatively analyzing the neural damage characteristics of each patient to establish customized rehabilitation strategies and predict prognosis. Therefore, this review provides valuable clinical insights for clinicians and holds significant importance as the systematic review for neural tracts causes of dysphagia using DTT that could significantly contribute to improving swallowing function recovery and overall quality of life for dysphagia patients. Still, future studies that would address the aforementioned limitations to further enhance understanding of neural tracts involved in dysphagia are recommended. Specifically, we propose proactively employing DTT to compare interventions, such as rTMS, thereby identifying optimal therapies that promote both functional recovery and the structural restoration of these crucial tracts.

## 5. Conclusions

This study investigated neural tracts associated with dysphagia using diffusion tensor tractography. The systematic review confirmed that the three neural tracts or pathways—the corticobulbar tract, vestibulospinal tract, and core vestibular pathway—were associated with dysphagia. Particularly, parameter values (i.e., FA and TV) of the CBT and VST showed negative correlations with dysphagia severity. That is, in clinical practice, utilizing DTT to identify neural tracts involved in dysphagia may allow more accurate prognostic prediction, facilitating better-targeted treatment planning and interventions for patients with dysphagia.

## Figures and Tables

**Table 1:** Details of the study using diffusion tensor tractography for stroke patients that cause dysphagia.

	Study	Study Design	Number of Patients	Type of Injury	Tract	Parameters	Outcomes
1	Jang et al. (2017) [33]	Case study	EG = 1 CG = 3	EG = left MCA territory CG = Normal	CBT	Imaging only (5 weeks, 9 weeks after onset)	Demonstrates the association of the recovery of injured CBT with the recovery of dysphagia using DTT.
2	Yeo et al. (2018) [39]	Cohort study	EG = 8 CG = 10	EG = lateral medullary syndrome CG = Normal	Core Vestibular Pathway (CVP)	FA, MD (average of 14 days: range 10–21 after onset)	Confirmed using DTT and presented that damage to the core vestibular pathway is correlated with causing dysphagia.
3	Jang et al. (2020) [34]	Cohort study	EG = 42 (A, B, C) CG = 22	EG = ICH (date of NGT removal) (A: within acute stage) (B: within 6 months after onset) (C: more than 6 months after onset) CG = normal	CBT	DTT: FA, TV (<6 weeks after onset; average of 21.39 ± 9.38 days) dysphagia: VFSS or GUSS	The evaluation of the CBT state using DTT would be helpful for the prognosis prediction of the NGT removal in the early stage of ICH.
4	Jang et al. (2020) [35]	Cohort study	EG = 20 (A, B) CG = 2	EG = LMI (date of NGT removal) (A: within 6 months after onset) (B: more than 6 months after onset) CG = normal	CBT	DTT: FA, TV (<6 weeks after onset; average of 16.0 ± 6.7 days) dysphagia: VFSS, PAS, FOIS	Confirmed using DTT and showed that the injury severity of the CBT in the affected hemisphere appeared to be related to a poor dysphagia prognosis following LMI.
5	Jang et al. (2020) [37]	Case report	EG = 1 CG = 6	EG = lateral medullary syndrome CG = normal	VST (medial, lateral)	DTT: FA, MD (2 weeks after onset)	Analysis of lateral VST using DTT can help evaluate lateral medullary syndrome patients with central vestibular disorder.
6	Jang et al. (2020) [38]	Cohort study	EG = 7 CG = 10	EG = lateral medullary syndrome of DIM infraction CG = normal	VST (medial, lateral) CST	DTT: FA, MD, TV (average of 14 days: range 10–21 after onset) motor function: FAC, MI, MBC	Analysis of the lateral VST using DTT can help evaluate lateral medullary syndrome patients with central vestibular signs such as dysphagia.
7	Wang et al. (2023) [36]	Pilot study	Group 1: 31 Group 2: 30	2 groups of stroke patients Group 1: high CBT integrity (5Hz rTMS, 1Hz rTMS, Sham rTMS) Group 2: low CBT integrity (5Hz rTMS, 1Hz rTMS, Sham rTMS)	CBT	DTT: FA dysphagia: SSA, PAS, DOSS	Confirmed using DTT and showed that higher rTMS was more effective than lower in treating dysphagia in patients with injured CBT after stroke

EG: experimental group, CG: control group, MCA: middle cerebral artery, DTT: diffusion tensor tractography, CBT: corticobulbar tract, CVP: core vestibular pathway, NGT: nasogastric tube, FA: fractional anisotropy, MD: mean diffusivity, ICH: intracerebral hemorrhage, VFSS: videofluoroscopic study, GUSS: Gugging swallowing screen, PAS: penetration aspiration scale, FOIS: functional oral intake scale, TV: tract volume, LMI: lateral medullary infarction, VST: vestibulospinal tract, CST: corticospinal tract, SSA: standardized swallowing assessment, DOSS: dysphagia outcome severity scale, FAC: functional ambulation category, MI: motricity index, MBC: modified Brunnstrom classification, rTMS: repetitive transcranial magnetic stimulation.

## Data Availability

No new data were created or analyzed in this study.

## References

[1] Yang X., Qiang Q., Li N., Feng P., Wei W., Hölscher C. (2022). Neuroprotective mechanisms of glucagon-like peptide-1-based therapies in ischemic stroke: An update based on preclinical research. Front. Neurol..

[2] Pega F., Náfrádi B., Momen N.C., Ujita Y., Streicher K.N., Prüss-Üstün A.M., Descatha A., Driscoll T., Fischer F.M., Godderis L. (2021). Global, regional, and national burdens of ischemic heart disease and stroke attributable to exposure to long working hours for 194 countries, 2000–2016: A systematic analysis from the WHO/ILO Joint Estimates of the Work-related Burden of Disease and Injury. Environ. Int..

[3] Kaneko N., Takeda H., Kudo M. (2020). Pathophysiology and Treatment of Stroke: Present Status and Future Perspectives. Int. J. Mol. Sci..

[4] Elendu C., Amaechi D.C., Elendu T.C., Ibhiedu J.O., Egbunu E.O., Ndam A.R., Ogala F., Ologunde T., Peterson J.C., Boluwatife A.I. (2023). Stroke and cognitive impairment: Understanding the connection and managing symptoms. Ann. Med. Surg..

[5] Feng W. (2023). Diagnosis of post-stroke dysphagia: Towards better treatment. Lancet Neurol..

[6] Song W., Wu M., Wang H., Pang R., Zhu L. (2024). Prevalence, risk factors, and outcomes of dysphagia after stroke: A systematic review and meta-analysis. Front. Neurol..

[7] Dziewas R., Michou E., Trapl-Grundschober M., Lal A., Arsava E.M., Bath P.M., Clavé P., Glahn J., Hamdy S., Pownall S. (2021). European Stroke Organisation and European Society for Swallowing Disorders guideline for the diagnosis and treatment of post-stroke dysphagia. Eur. Stroke J..

[8] Son Y.S., Min K.C., Woo H.S. (2022). Effect of oral motor facilitation technique (OMFT) and neuromuscular electrical stimulation (NMES) applied to a patient with Wallenberg’s syndrome: A case study. Ther. Sci. Rehabil..

[9] Zhang L., Tang X., Wang C., Ding D., Zhu J., Zhou Y., Diao S., Kang Y., Cai X., Li C. (2021). Predictive Model of Dysphagia and Brain Lesion-Symptom Mapping in Acute Ischemic Stroke. Front. Aging Neurosci..

[10] Martino R., Foley N., Bhogal S., Diamant N., Speechley M., Teasell R. (2005). Dysphagia after stroke: Incidence, diagnosis, and pulmonary complications. Stroke.

[11] George R.G., Jagtap M. (2022). Impact of swallowing impairment on quality of life of individuals with dysphagia. Indian J. Otolaryngol. Head. Neck Surg..

[12] Karisik A., Dejakum B., Moelgg K., Komarek S., Toell T., Mayer-Suess L., Pechlaner R., Kostner S., Sollereder S., Kiechl S. (2024). Association between dysphagia and symptoms of depression and anxiety after ischemic stroke. Eur. J. Neurol..

[13] Zeng H., Zeng X., Xiong N., Wang L., Yang Y., Wang L., Li H., Zhao W. (2024). How stroke-related dysphagia relates to quality of life: The mediating role of nutritional status and psychological disorders, and the moderating effect of enteral nutrition mode. Front. Nutr..

[14] Kumar S., Marchina S., Langmore S., Massaro J., Palmisano J., Wang N., Searls D.E., Lioutas V., Pisegna J., Wagner C. (2022). Fostering eating after stroke (FEASt) trial for improving post-stroke dysphagia with non-invasive brain stimulation. Sci. Rep..

[15] Lee W.H., Lim M.H., Seo H.G., Seong M.Y., Oh B.M., Kim S. (2020). Development of a novel prognostic model to predict 6-month swallowing recovery after ischemic stroke. Stroke.

[16] Fandler S., Gattringer T., Eppinger S., Doppelhofer K., Pinter D., Niederkorn K., Enzinger C., Wardlaw J.M., Fazekas F. (2017). Frequency and predictors of dysphagia in patients with recent small subcortical infarcts. Stroke.

[17] Flowers H.L., AlHarbi M.A., Mikulis D., Silver F.L., Rochon E., Streiner D., Martino R. (2017). MRI-based neuroanatomical predictors of dysphagia, dysarthria, and aphasia in patients with first acute ischemic stroke. Cerebrovasc. Dis. Extra.

[18] Wilmskoetter J., Bonilha L., Martin-Harris B., Elm J.J., Horn J., Bonilha H.S. (2019). Mapping acute lesion locations to physiological swallow impairments after stroke. NeuroImage Clin..

[19] Fandler S., Gattringer T., Pinter D., Pirpamer L., Borsodi F., Eppinger S., Niederkorn K., Enzinger C., Fazekas F. (2018). Dysphagia in supratentorial recent small subcortical infarcts results from bilateral pyramidal tract damage. Int. J. Stroke.

[20] Baltzer P.A., Mann R.M., Iima M., Sigmund E.E., Clauser P., Gilbert F.J., Martincich L., Partridge S.C., Patterson A., Pinker K. (2020). Diffusion-weighted imaging of the breast-a consensus and mission statement from the EUSOBI International Breast Diffusion-Weighted Imaging working group. Eur. Radiol..

[21] Mori S., Van Zijl P.C. (2002). Fiber tracking: Principles and strategies—A technical review. NMR Biomed..

[22] Kim M.S., Moon B.S., Ahn J.Y., Shim S.S., Yun J.M., Joo M.C. (2022). Elucidating the mechanisms of post-stroke motor recovery mediated by electroacupuncture using diffusion tensor tractography. Front. Neurol..

[23] Beaulieu C. (2002). The basis of anisotropic water diffusion in the nervous system—A technical review. NMR Biomed..

[24] Seo J.P., Jang S.H. (2013). Different characteristics of the corticospinal tract according to the cerebral origin: DTI study. Am. J. Neuroradiol..

[25] Christidi F., Tsiptsios D., Fotiadou A., Kitmeridou S., Karatzetzou S., Tsamakis K., Sousanidou A., Psatha E.A., Karavasilis E., Seimenis I. (2022). Diffusion Tensor Imaging as a Prognostic Tool for Recovery in Acute and Hyperacute Stroke. Neurol. Int..

[26] Zhang F., Daducci A., He Y., Schiavi S., Seguin C., Smith R.E., Yeh C.-H., Zhao T., O’Donnell L.J. (2022). Quantitative mapping of the brain’s structural connectivity using diffusion MRI tractography: A review. NeuroImage.

[27] Kamagata K., Andica C., Uchida W., Takabayashi K., Saito Y., Lukies M., Hagiwara A., Fujita S., Akashi T., Wada A. (2024). Advancements in Diffusion MRI Tractography for Neurosurgery. Investig. Radiol..

[28] Laurell A.A., Mak E., O’Brien J.T. (2025). A systematic review of diffusion tensor imaging and tractography in dementia with Lewy bodies and Parkinson’s disease dementia. Neurosci. Biobehav. Rev..

[29] Kumar A.A., Zhang J.J., Pillay R., Keong N.C. (2025). Utility of diffusion tensor imaging and tractography (DTI/DTT) in the surgical resection of intramedullary spinal cord tumors: A scoping review. Eur. Spine J..

[30] Alvar A., Hahn Arkenberg R., McGowan B., Cheng H., Malandraki G.A. (2021). The role of white matter in the neural control of swallowing: A systematic review. Front. Hum. Neurosci..

[31] Higgins J.P.T., Thomas J., Chandler J. (2023). Cochrane Handbook for Systematic Reviews of Interventions.

[32] Page M.J., McKenzie J.E., Bossuyt P.M., Boutron I., Hoffmann T.C., Mulrow C.D., Shamseer L., Tetzlaff J.M., Akl E.A., Brennan S.E. (2021). The PRISMA 2020 statement: An updated guideline for reporting systematic reviews. BMJ.

[33] Jang S.H., Kim J., Seo Y., Kwak S.Y. (2017). Recovery of an injured corticobulbar tract in a patient with stroke: A case report. Medicine.

[34] Jang S.H., Kwak S.Y., Chang C.H., Jung Y.J., Kim J., Kim S.H., Kim J.Y. (2020). Prognostic prediction of dysphagia by analyzing the corticobulbar tract in the early stage of intracerebral hemorrhage. Dysphagia.

[35] Jang S.H., Lee J., Kim M.S. (2020). Dysphagia prognosis prediction via corticobulbar tract assessment in lateral medullary infarction: A diffusion tensor tractography study. Dysphagia.

[36] Wang L., Wang F., Lin Y., Guo X., Wang J., Liu J., Feng C., Xu S., Wang Y., Gao C. (2023). Treatment of post-stroke dysphagia with repetitive transcranial magnetic stimulation based on the bimodal balance recovery model: A pilot study. J. Integr. Neurosci..

[37] Jang S.H., Oh S., Yeo S.S. (2020). Lateral medullary syndrome following injury of lateral vestibulospinal tract: Diffusion tensor imaging study. J. Stroke Cerebrovasc. Dis..

[38] Jang S.H., Park G.Y., Cho I.H., Yeo S.S. (2020). Injury of the lateral vestibulospinal tract in a patient with the lateral medullary syndrome: Case report. Medicine.

[39] Yeo S.S., Jang S.H., Kwon J.W. (2018). Lateral medullary syndrome following injury of the vestibular pathway to the core vestibular cortex: Diffusion tensor imaging study. Neurosci. Lett..

[40] Broadbent W.H. (1866). On a case of right hemiplegia, with deviation of the eyes to the left, and aphasia. Lancet.

[41] Gowers W.R. (1898). A Manual of Diseases of the Nervous System.

